# Feasibility and safety of surgery for coronary artery disease and gastrointestinal malignancy: a propensity score matching analysis

**DOI:** 10.1186/s40001-026-03842-x

**Published:** 2026-01-10

**Authors:** Zhengjie Zhang, Mingkui Zhang, Yuheng Jiang, Li Zhao, Hui Xue, Guoshan Yang, Lixin Fan, Yanbin Shao, Qingyu Wu

**Affiliations:** 1 Heart Center, First Hospital of Tsinghua University, Tsinghua University, No.6, 1 st Jiuxiaoqiao, Chaoyang District, Beijing, 100016 China; 2 Department of General Surgery, First Hospital of Tsinghua University, Tsinghua University, Beijing, China; 3 Department of Anesthesiology, First Hospital of Tsinghua University, Tsinghua University, Beijing, China

**Keywords:** Coronary artery disease, Gastrointestinal tumors, Coronary artery bypass grafting, Surgical treatment, Feasibility

## Abstract

**Background:**

The treatment strategy for severe coronary artery disease (CAD) combined with gastrointestinal malignant tumors remains controversial. This study aimed to explore the feasibility and safety of surgical treatment for severe CAD and gastrointestinal malignant tumors.

**Materials and methods:**

From January 2018 to May 2025, eight patients with severe CAD and gastrointestinal cancer underwent surgical treatment at our institution. A propensity score matching analysis was performed to balance baseline characteristics between the CABG group (group 1) and the CABG + tumor resection (TR) group (group 2). The surgical procedures and preliminary outcomes of both groups were compared, along with survival status and cardiac adverse events during follow-up.

**Results:**

Eight cases of gastrointestinal malignant tumors combined with CAD were identified, with seven patients undergoing off-pump CABG and gastrointestinal cancer radical resection in single stage or staged (one case received chemotherapy). 67 cases of isolated off-pump CABG were subjected to propensity score matching analysis, with a 1:1 matching ratio to compare perioperative outcomes between the two groups. Patients were followed up for postoperative cardiac adverse events, tumor recurrence, and survival time. Except for surgical duration, there were no significant differences between the two groups in 24-h drainage, intraoperative blood transfusion, ventilator support time, ICU stay duration, postoperative hospital stay duration, perioperative myocardial infarction, stroke, and ARDS (*p* > 0.05). All patients were discharged in good condition, without perioperative infections, or anastomotic leaks. At follow-up ranging from 2 to 45 months (mean 19 months), one colorectal cancer patient died suddenly during chemotherapy 15 months postoperatively, and one gastric lymphoma patient died from lymphoma recurrence 41 months postoperatively. The remaining patients did not experience tumor recurrence or metastasis, and no cardiac adverse events occurred.

**Conclusions:**

For patients with severe CAD and gastrointestinal tumors, Off-Pump CABG and gastrointestinal cancer radical surgery is safe and feasible. Selecting appropriate patients for surgery can achieve good clinical outcomes.

## Introduction

The incidence of coronary artery disease (CAD) combined with malignant tumors has increased significantly due to common risk factors. Malignant tumors also lead to a significant increase in the risk of cardiovascular mortality and non-fatal events [1, 2]. For patients with severe CAD, the incidence of cardiac adverse events is very high during the perioperative period of tumor resection surgery [3]. The coexistence of CAD and gastrointestinal malignant tumors often leads to treatment conflicts. Coronary artery disease requires antiplatelet therapy to maintain the efficacy of revascularization, while malignant tumor treatment must avoid the risk of bleeding [4–6]. Therefore, the high risk of cardiac surgery must be balanced with the urgency of malignant tumor progression, and treatment decisions must consider both the efficacy and safety of surgery.

Following drug-eluting stent implantation, antiplatelet therapy is required for 6–12 months [7–9]. In contrast, CABG allows discontinuation of antiplatelet therapy within 2–4 weeks, making it a preferable option for cardiac revascularization in patients with malignant tumors [10]. However, factors such as survival time, perioperative risks, and surgical strategy selection may influence the surgeon’s decision-making process. Additionally, there remains conflicting evidence regarding the treatment of CAD in patients with tumors. Some studies suggest that simultaneous CABG and tumor resection surgery may be feasible [11].

There have been few studies on combined surgery for gastrointestinal malignant tumors and CABG, and the incidence of perioperative complications is high [12]. This retrospective study used propensity score matching analysis to evaluate the safety and feasibility of CABG and radical surgery for gastrointestinal malignant tumors.

## Methods

### Patient selection

This retrospective study was approved by the Ethics Committee of the First Hospital of Tsinghua University (No.(R)2025-057-01). Written informed consent was obtained for all patients underwent surgical treatment. The Ethics Committee waived the requirement for written informed consent for data publication. This report reviewed patients who underwent CABG and gastrointestinal tumor surgery in the clinical electronic medical record system of the First Hospital of Tsinghua University from January 2018 to May 2025. A propensity score matching analysis was performed to balance baseline characteristics between the CABG group (group 1) and the CABG + tumor resection (TR) group (group 2). Preoperative assessment was performed using coronary angiography, echocardiography, gastrointestinal endoscopy, positron emission tomography (PET) or computed tomography (CT) imaging, or PET/CT combined imaging.

### Inclusion criteria

Indications: CABG is primarily indicated for severe stenosis at the origin of the left anterior descending artery, three-vessel disease, left main coronary artery disease, or concomitant three-vessel disease. Additionally, gastrointestinal tumor surgery must meet the following criteria: (i) based on preoperative tumor, lymph node, and metastasis (TNM) staging, if the tumor is in an early stage (Stage I or II) or suitable for curative surgery, with an estimated prognosis of > 1 year, and/or (ii) CAD impedes oncological treatment (surgery or chemotherapy), and such treatment is beneficial for improving the patient’s prognosis [11]. Exclusion criteria: tumor metastasis; other concomitant cardiovascular diseases; organ failure; and other surgical contraindications.

### Surgical strategy 

A multidisciplinary team collaborates to develop a treatment plan based on cardiac function, coronary angiography, tumor TNM staging, comorbidities (hypertension, diabetes), and overall health status. If medically indicated, preoperative adjuvant chemotherapy may be administered.

Surgical sequence for concurrent surgery: Typically, myocardial ischemia is addressed first, followed by tumor resection, to reduce cardiac load and avoid interference from anticoagulant medications.

Interval between staged surgeries: For patients who cannot tolerate concurrent surgery or opt for staged surgery, tumor surgery can be performed 2–6 weeks after CABG.

### Surgical technique

Off-pump CABG is performed via a left anterior lateral thoracotomy, using the left internal mammary artery (LIMA) to revascularize the left anterior descending artery (LAD), or via a median sternotomy for revascularization [11]. The left anterior lateral thoracotomy approach involves a left anterior fourth intercostal thoracotomy, with the left chest elevated to a 20–30°supine position. The left internal thoracic artery is harvested using the Cardio Surgical System (Cardio Surgical Limited, Galway, Ireland), which provides a visual pathway to expose the entire length of the left internal thoracic artery. A coronary artery target vessel local stabilization device (Medtronic, Inc., Minneapolis, MN, USA) is used for off-pump CABG procedures, serving to compress and secure the vessel, with the proximal coronary artery occluded using silicone tape. The distal coronary artery is anastomosed using 7–0 polypropylene sutures, and the proximal end is sutured with 6–0 polypropylene sutures. The remaining grafts are from the great saphenous vein.

Following completion of the coronary artery bypass grafting procedure, close the surgical incision on the chest. Re-disinfect the abdominal surgical site and drape it with sterile surgical drapes.

Colon and rectal surgery are performed in the supine position or modified lithotomy position. After establishing the observation port, the laparoscope is used to directly visualize and place the main operative ports and auxiliary operative ports, followed by insertion of the trocar. Following the standard procedure for total colectomy/mesorectal excision. The cutting edge of colon cancer surgery is about 10 cm away from the tumor, while the cutting edge of rectal cancer is more than 5 cm away from the tumor. After resection, an appropriate anastomosis method is selected based on the situation.

Gastric cancer radical resection surgery is performed in the supine position with legs abducted. A laparoscope is placed below the umbilicus to examine for abdominal metastases. After establishing the operative channel, a standard laparoscopic distal gastric D2 radical resection is performed.

### A propensity score matching

**Fig. 1:**
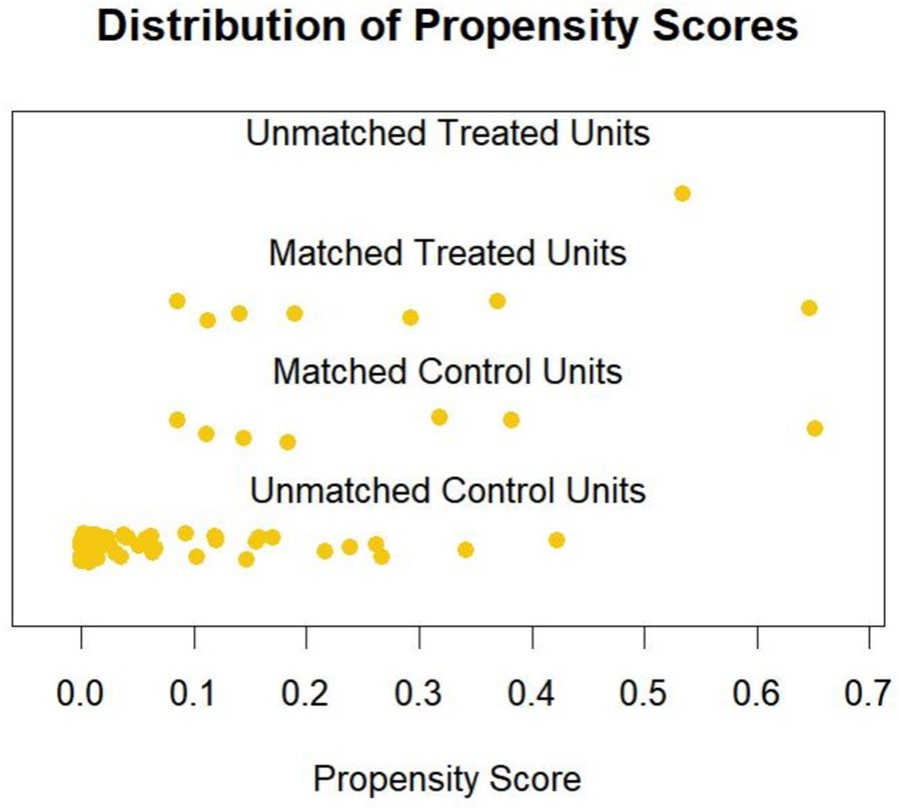
Distribution of propensity score

Sixty-seven cases of isolated off-pump CABG were subjected to propensity score matching analysis, with a 1:1 matching ratio (7:7) to compare perioperative outcomes between the two groups. Matching factors included gender, age, body mass index, hypertension, diabetes, hyperlipidemia, smoking history, and left ventricular ejection fraction. Baseline data before and after matching are shown in Table 1. The distribution of scores after matching is shown in Fig. 1.

**Table 1:** Baseline characteristics of the patients before and after matching

Variable	Before matching			After matching		
	Group 1 (*n* = 67)	Group 2 (*n* = 8)	*P* Value	Group 1 (*n* = 7)	Group 2 (*n* = 7)	*P* Value
Age (mean ± SD)	59.66 ± 9.73	64.50 ± 6.76	0.176	62.29 ± 6.75	63.29 ± 6.29	0.779
Gender (n, %)			1.000			1.000
Male	59(88.1)	7 (87.5)		6 (85.7)	6 (85.7)	
Female	8 (11.9)	1 (12.5)		1 (14.3)	1 (14.3)	
BMI (mean ± SD)	25.73 ± 3.78	27.06 ± 2.93	0.342	28.64 ± 3.25	27.20 ± 3.14	0.416
Smoking (n, %)	29 (43.3)	3 (37.5)	1.000	3 (42.9)	2 (28.6)	1.000
Diabetes (n, %)	33 (49.3)	6 (75.0)	0.316	5 (71.4)	5 (71.4)	1.000
Hypertension (n, %)	38 (56.7)	7 (87.5)	0.194	6 (85.7)	6 (85.7)	1.000
Dyslipidemia (n, %)	52 (77.6)	5 (62.5)	0.611	5 (71.4)	4 (57.1)	1.000
NYHA class (n, %)			0.427			1.000
I	22 (32.8)	4 (50.0)		4 (57.1)	4 (57.1)	
II	36 (53.7)	4 (50.0)		3 (42.9)	3 (42.9)	
III	9 (13.4)	0 (0.0)		0 (0.0)	0 (0.0)	
LVEF (mean ± SD)	58.88 ± 6.66	55.62 ± 11.92	0.239	56.71 ± 6.37	59.14 ± 7.08	0.513

*CABG* coronary artery bypass grafting; *TR* tumor resection; *SD* standard deviation; *SMD* standardized mean difference; *NYHA* New York Heart Association Classification; *BMI* body mass index; *LVEF* left ventricular ejection fraction

### Follow-up

Follow up with patients through outpatient visit records and telephone calls, recording clinical symptoms, electrocardiograms, echocardiograms, chest X-rays, current survival status, and antitumor treatment status. All patients were followed up for mean 19 months.

### Statistical analysis

Statistical analysis was performed using R Software v.4.4.0 (The R Project for Statistical Computing, www.r-project.org). Continuous variables were expressed as mean ± standard deviation (SD). Comparisons between groups were performed using an independent samples t-test. Categorical variables were expressed as counts (n). Comparisons between groups were performed using the chi-square test or Fisher’s exact test. Propensity score matching was performed using the MatchIt package. A *P*-value < 0.05 was considered statistically significant.

## Results

### Patient characteristics

Eight patients were enrolled, including seven males and one female, with ages ranging from 55 to 73 years (64.5 ± 6.76 years). Among the eight patients, seven underwent radical surgery for gastrointestinal malignant tumors, including two cases of gastric cancer, three cases of colorectal cancer, two cases of rectal cancer, and one case of advanced lymphoma in the stomach. The most common symptoms associated with gastrointestinal malignant tumors are anemia, weight loss, and gastrointestinal bleeding. All patients underwent coronary angiography in group 2, revealing 1 case of left anterior descending artery lesion, 1 case of double-vessel disease, and 6 cases of triple-vessel coronary artery disease.

### Surgical outcomes

The average graft numbers were 2 (2 ± 1.06). The three simultaneous surgeries were performed, including two patients undergoing off-pump CABG and colorectal cancer resection, and one patient undergoing off-pump CABG and gastric cancer resection. Four cases of staged surgery were performed, including one patient who underwent off-pump CABG and gastrectomy for gastric cancer, one patient who underwent off-pump CABG and colorectal cancer resection, and two patients who underwent off-pump CABG and rectal cancer resection. One patient with gastric lymphoma continued chemotherapy after off-pump CABG surgery.

All patients underwent histological diagnosis. Seven patients were diagnosed with adenocarcinoma, and one patient was diagnosed with gastric lymphoma. TNM staging: 1 case of stage 0, 2 cases of stage 1, 3 cases of stage 2, and 2 cases of stage 3. One patient with rectal cancer, staged as stage 3, underwent neoadjuvant chemotherapy, resulting in tumor shrinkage, followed by CABG, and then underwent radical resection for rectal cancer 3 months postoperatively.

### Propensity score matching

The baseline characteristics of patients before and after propensity score matching are summarized in Table 1. There were no significant differences between the two groups in terms of age, gender, body mass index, diabetes, hypertension, hyperlipidemia, NYHA functional class, and left ventricular ejection fraction (*p* > 0.05).

There were no significant differences between the two groups in terms of hospital stay, ICU stay, blood transfusion volume, postoperative 24-h drainage volume, ventilator support time, perioperative myocardial infarction, stroke, or ARDS (*p* > 0.05), except for surgical time (Table 2).

**Table 2:** Intraoperative and postoperative variables

Variable	Group 1 (*n* = 7)	Group 2 (*n* = 7)	*P* Value
Surgery time (min)	339.00 ± 53.42	575.00 ± 186.32	0.007
Drainage of 24 h (ml)	303.71 ± 75.63	320.00 ± 166.96	0.818
Postoperative length of stay (day)	10.71 ± 5.15	11.43 ± 5.74	0.811
Intraoperative blood transfusion (unit)	0.00 ± 0.00	0.86 ± 1.18	0.079
ICU > 48 h (*n*, %)	3 (42.9)	2 (28.6)	1.000
Mechanical ventilation > 8 h (*n*, %)	2 (28.6)	2 (28.6)	1.000
Perioperative period MI (*n*, %)	0 (0.0)	0 (0.0)	1.000
Perioperative period stroke (*n*, %)	0 (0.0)	0 (0.0)	1.000
Perioperative period ARDS (*n*, %)	0 (0.0)	0 (0.0)	1.000

*ICU* intensive care unit; *MI* myocardial infarction; *AEDS* acute respiratory distress syndrome

### Follow-up results

All patients were followed up for 2–45 months (mean 19 months). One patient with gastric lymphoma died 41 months after surgery due to lymphoma recurrence. One patient with colon cancer died suddenly 15 months after surgery, following chemotherapy. No adverse events such as recurrent angina, myocardial infarction, fatal arrhythmia, or heart failure occurred in the remaining patients during follow-up.

## Discussion

Gastrointestinal malignant tumors are more common in patients with coronary artery disease (CAD). The higher prevalence of colorectal tumors in CAD-positive patients may be due to the many shared risk factors between CAD and colorectal tumors, such as advanced age, male gender, smoking, obesity, and diabetes [2, 13]. For patients with advanced malignant tumors and left main coronary artery or three-vessel disease, coronary artery bypass grafting can reduce the incidence of adverse cardiac events during tumor surgery. The main finding of this study is that combining coronary artery bypass grafting with radical tumor surgery in patients with gastrointestinal malignant tumors and coronary heart disease can achieve good results and is an effective treatment approach.

For patients with advanced gastrointestinal malignant tumors, there remains controversy regarding the appropriateness of coronary artery bypass surgery. Due to the varying stages and differentiation levels of tumors, literature on this topic is scarce [14]. For patients with gastrointestinal tumors complicated by severe coronary artery disease, surgeons face significant challenges in making decisions. We believe the following factors are crucial for surgeons’ decision-making: (ⅰ) The location, extent, biological characteristics, and TNM staging of the tumor, as well as the anticipated benefits or survival time; (ⅱ) The severity of myocardial ischemia and its impact on cardiac risk during tumor surgery; (ⅲ) The comprehensive assessment of the patient and the risk of perioperative infection if concurrent or staged surgery is performed; (ⅳ)The contradiction between preoperative acute or chronic bleeding from severe ulceration of the gastrointestinal tumor and antiplatelet therapy. Similar to previous studies [14, 15], we believe that for early-stage gastrointestinal cancer patients (stages I-II), undergoing CABG can yield encouraging long-term outcomes; for patients with advanced TNM staging, if further treatment can improve cancer-related prognosis, myocardial revascularization should still be actively pursued. Cardiac surgery is contraindicated only when the cancer is at an advanced stage and no treatment options can extend survival or improve quality of life.

There is still some controversy regarding whether CABG and malignant tumor resection should be performed as a single procedure or in stages [11, 16]. Concurrent CABG and tumor resection offer advantages such as high cost-effectiveness, reduced trauma from two separate surgeries, no delay in tumor surgery, and minimal psychological impact on patients [17, 18]. A study found that concurrent CABG and lung tumor resection is a safe and effective method, allowing for early completion of lung surgery and avoiding complications caused by delayed surgery [19]. However, concurrent surgery also has drawbacks such as prolonged surgical duration and increased risk of infection. Komokata et al. [12] reported on 15 cases of simultaneous gastrointestinal tumor and cardiac surgery, with a postoperative complication rate of 33.3%, including stroke, pneumonia, bleeding, and hyperbilirubinemia, with one surgical death. In this group, there were no perioperative angina pectoris, perioperative deaths, myocardial infarctions, or other cardiac events. Therefore, we believe that coronary artery bypass grafting concurrently or sequentially with radical resection of gastrointestinal tumors is feasible and safe. The choice between concurrent or sequential surgical strategies primarily depends on the severity of ischemic heart disease, the extent of tumor lesions, and the surgeon’s experience.

The long-term outcomes of coronary artery bypass grafting (CABG) and radical surgery for gastrointestinal tumors have not yet been reported. Studies have found that off-pump CABG combined with lung resection does not increase the incidence of cardiac adverse events, but surgical complications are significantly increased [18]. During the follow-up period, no cardiovascular adverse events such as recurrent angina, acute myocardial infarction, fatal arrhythmias, or heart failure occurred in this group of patients. Therefore, for patients with early-stage gastrointestinal tumors or those with a projected long survival time, we recommend complete myocardial revascularization, which can effectively prevent major adverse cardiac events during the perioperative period or tumor treatment process. For cancer patients who may achieve good outcomes and those with CAD who can be treated surgically, the safety of coronary artery bypass grafting combined with tumor resection surgery is reliable, and long-term survival rates are closely related to tumor stage [20].

The limitations of this study include the fact that it is a small-sample retrospective study, and the bias in this study is undeniable; more clinical data on surgical treatment for cases of gastrointestinal malignant tumors combined with CAD are needed. Secondly, due to the small sample size, further classification studies are challenging. The value of tumor TNM staging in the selection of treatment strategies for patients with gastrointestinal malignant tumors combined with coronary artery disease needs further study.

## Conclusions

In summary, simultaneous or staged gastrointestinal tumor radical surgery with coronary artery bypass grafting has high feasibility and safety. Selecting appropriate patients for surgery can achieve good clinical outcomes.

## Data Availability

Data are available on reasonable request to the corresponding author.
